# Correction: Batwa Indigenous Peoples forced eviction for “Conservation”: A qualitative examination on community impacts

**DOI:** 10.1371/journal.pgph.0002413

**Published:** 2023-09-14

**Authors:** The PLOS Global Public Health Staff

## Notice of Republication

This article was republished on August 21, 2023, to correct capitalization in the title. The publisher apologizes for the errors. Please download this article again to view the correct version. The originally published, uncorrected article and the republished, corrected articles are provided here for reference.

## Supporting information

S1 File Originally published, uncorrected article. (PDF)

S2 File Republished, corrected article. (PDF)
