# Supplementary material for: Correction: Batwa Indigenous Peoples forced eviction for “Conservation”: A qualitative examination on community impacts
Source: PLOS Glob Public Health. 2023 Sep 14;3(9):e0002413. doi: 10.1371/journal.pgph.0002413 (PMC10501623; doi:10.1371/journal.pgph.0002413)
Supplement: S2 File — (PDF) [file pgph.0002413.s002.pdf]

PLOS GLOBAL PUBLIC HEALTH

RESEARCH ARTICLE

Batwa Indigenous Peoples forced eviction for "Conservation": A qualitative examination on community impacts

Sylvia Kokunda[1☯], Haven Nahabwe[2☯], Jeremiah Nahamya[1], Samari Niwamanya[1], Ronald Mazirwe[1], Samrawit Gougsa[3,4], Elizabeth Kemigisha[5,6], Nicole Redvers[7☯]*

1 Action for Batwa Empowerment Group, Kanungu District, Uganda, **2** Bwindi Community Hospital, Community Health and Batwa Department, Kanungu, Uganda, **3** Minority Rights Group, London, United Kingdom, **4** The Hub at Wellcome Collection, London, United Kingdom, **5** Department of Human Development and Relational Sciences, Faculty of Interdisciplinary Studies, Mbarara University of Science and Technology, Mbarara, Uganda, **6** African Population and Health Research Center, Nairobi, Kenya, **7** Schulich School of Medicine & Dentistry, Western University, London, Ontario, Canada

☯ These authors contributed equally to this work.
* nredvers@uwo.ca

Check for updates

OPEN ACCESS

Citation: Kokunda S, Nahabwe H, Nahamya J, Niwamanya S, Mazirwe R, Gougsa S, et al. (2023) Batwa Indigenous Peoples forced eviction for "Conservation": A qualitative examination on community impacts. PLOS Glob Public Health 3(8): e0002129. https://doi.org/10.1371/journal.pgph.0002129

Editor: Mathew Sunil George, University of Canberra, AUSTRALIA

Received: April 28, 2023

Accepted: July 6, 2023

Published: August 16, 2023

Copyright: © 2023 Kokunda et al. This is an open access article distributed under the terms of the Creative Commons Attribution License, which permits unrestricted use, distribution, and reproduction in any medium, provided the original author and source are credited.

Data Availability Statement: Due to Indigenous ethical considerations and Indigenous data sovereignty, any data and materials associated with this research will not be available unless additional relevant Indigenous ethics agreements are in place with the local community. Please contact Tumuhimbise Ishmael from the Action for Batwa Empowerment Group at tumuhimbiseishmael@gmail.com for any data requests pertaining to this study.

Abstract

In 1991, the Ugandan government formally established National Parks within the ancestral homelands of the Batwa Peoples. No consultation was carried out with local Batwa communities, and they were consequently forcibly evicted from their Forest home. With this, we sought to better understand the impacts of forced Land eviction through the lens of solastalgia. Nineteen semi-structured interviews were carried out with adult Batwa Peoples of varying age and gender in Uganda from August to November 2022. Interviews were transcribed verbatim, and thematic analysis was carried out on the interview transcripts to identify themes from the initial codes. Four overarching themes were identified, including: 1) Our love and connection with the Forest; 2) What was left in the Forest when we were evicted; 3) What eviction from the Forest did to us as Batwa Peoples; and 4) Batwa People's Landback and returning to the Forest ('Indigenous Lands back into Indigenous hands'). As movement towards the global "30 by 30" conservation agenda occurs, we urge researchers, policy makers, and leaders to listen to the voices of Indigenous Peoples like the Batwa with a key focus on Landback and movement towards a clearer understanding and appreciation of the impacts of Western conservation agendas on Indigenous Peoples globally.

Introduction

> "*The only thing that has displaced more people around the world than war is wildlife conservation. For Indigenous Peoples, the consequences are the same*" [1].

In the early 1990s, the Ugandan government formally established the Bwindi Impenetrable National Park [2]. No consultation was carried out with local Batwa Indigenous Peoples whose homes were in the Forests, and Batwa Peoples were consequently forcibly evicted from their Forest home [3]. Since that time, the World Bank under its Global Environment Facility

Funding: This work was supported by the Wellcome Trust (#220767/Z/20/Z to SG). The funders had no role in study design, data collection and analysis, decision to publish, or preparation of the manuscript.

Competing interests: The authors have declared that no competing interests exist.

(GEF), had established a Trust fund with the Ugandan government to manage the park with 60% of funding supposedly earmarked for local communities (i.e., The Mgahinga and Bwindi Impenetrable Forest Conservation Trust [MBIFCT]) [3].

Despite numbers of conservation groups, international organizations, and local governments citing impressive collaborative conservation work with local communities, the realities on the ground in Batwa communities were and are a far cry from the outwardly portrayed narratives [2, 4–7]. With clear guidelines for Batwa community involvement and decision making in the Trust fund, and clear, delineated funds meant to support local communities, support was and is still not reaching the ground [8]. Despite these clear breaches of international agreement, there is a lack of conservation voices standing up for Batwa communities to ensure that colonial and paternalistic systems that have continued to marginalize and exclude them from decision making and funding availability is rectified. "The emphasis on the conservation of natural resources and promotion of the tourist industry. . ." has clearly overridden all other interests despite the cost to Batwa Indigenous Peoples [3].

With clear socioeconomic deprivation, outright forced food insecurity due to Land displacement, prejudice and racism, and a lack of respect for the human rights of Batwa Peoples under the United Nation Declaration on the Rights of Indigenous Peoples (UNDRIP) [9] being honored (e.g., UNDRIP articles 1 and 21.1), there has been a clear lack of voice from Batwa communities being amplified in wider conservation dialogues [2, 5, 8, 10, 11]. There have been numerous and repeated case studies and research from "outside" actors and groups in order to "solve the problems" of the Batwa; however, missing from these public-facing dialogues is Batwa leadership and their direct voice not being diluted through external research narratives that have often failed to situate Batwa ways of knowing and being.

Studies and narratives that have highlighted the impacts of the forced eviction from traditional homelands (including the lack of access to forests and foods) by Batwa Peoples for Batwa Peoples are completely lacking. In particular, solastalgia or distress that is produced by environmental change [12] has been artificially induced through forced displacement of Batwa Peoples. Although socioeconomic and development impacts are relevant, the mental and emotional impacts from forced Land evictions of Indigenous Peoples in Africa have been overlooked and rarely examined. There has never been "Batwa-led research" carried out to date to better understand the individual and collective impacts of the land eviction process on their community through a holistic lens. With this, the overarching aim of the current research was to better understand solastalgia in the context of the forced eviction of Batwa Peoples from their Forest home.

Positionality

"Nothing about us, without us" in the context of Indigenous Peoples' related research is increasingly expected from both an ethical perspective as well as from a rights perspective [13–15]. Given this, we position ourselves here in this research to situate our context to the work and to the community. Six out of the eight co-authors are living within Uganda (SK, HN, JN, SN, RM, EK), with the lead author (SK), being from the Batwa Indigenous community. One co-author is based out of the UK providing capacity building assistance (SG), and one co-author is an invited Indigenous health scholar, who is a member of the Deninu K'ue First Nation (Canada) providing research technical support.

Materials and methods

Research questions

The present study was entirely Batwa community directed. The research questions were co-developed by request from the Batwa community members with the other team members to

ensure the study was done in a way that supported community-level priorities and understanding. The questions posed were: 1) What have been the impacts on our Batwa community due to the forced eviction from our Forest?; and 2) What does the Forest mean to us as Batwa Peoples, and how do we depend on the Forest?

Overall study design

A qualitative study was designed and led by Batwa community members in concert with local organizations and international partnerships. Indigenous Peoples have been significantly marginalized within research and practice leading to data extraction and narratives often being developed and shared by outsiders [16–18]. Given this, a decolonial theoretical and applied approach was engaged ensuring a "collaborative process of naturalizing Indigenous intent, interactions, and processes and making them evident to transform spaces, places, and heart" [19].

Interviews were determined to be the most appropriate method to share information situated in the context of "deep listening" [20], while allowing participants to converse in the local Rukiga language. Research team members outside of the Batwa community were engaged in supportive roles as defined by Batwa community members to ensure an Indigenous research methodology was honoured (SG, NR). The use of Indigenous research methodologies does not preclude the ability to carry out rigorous community-led research, so standards for reporting qualitative research (SRQR) [21] were followed.

Setting

The research was carried out in Batwa settlements in southwestern Uganda representing Bwanya, Kitahurira, Byumba, Kitariro, Kihembe, Kebiremu, Bikuto, Mukongoro, Buhoma, and Byumba.

Recruitment and consent

The research received full ethics board approvals from the two required bodies to carry out this work including: 1) Mbara University of Science and Technology (MUST) Research Ethics Committee (#MUST-2021-322), and 2) St. George's Research Ethics Committee (#2021.0230).

Purposive sampling was engaged through established local networks of the research team to ensure maximum variation of participants across the various Batwa settlements as well as to ensure gender balance. Age was also considered with the intent to have members of three specific groups represented including: 1) those who lived in the Forest before the forced land eviction (e.g., current Elders); 2) those who were born after the forced land eviction (i.e., lived during the eviction as a young person); and 3) those born after the forced land eviction. Only adult members (>18yo) of the Batwa community in Uganda were included in the study sample.

A local research assistant visited the various Batwa settlements in person to provide information to potential participants on the research being undertaken. No other recruitment methods were used as the in-person visits were determined to be the most feasible for passing on information about the study given the local context. Participant information sheets were available in both English and the local Rukiga language to ensure potential participants had the opportunity to consider the study and ask questions in the language that was most comfortable for them. At a later date, two research assistants (one male and one female) came back to the communities, and the consent form was reviewed verbally with interested participants while ensuring they had the time needed to decide on participation. On this second visit of the research team, those who wanted to proceed with participation gave informed consent. If a

participant needed to travel to participate outside of their home area, a snack and tea were provided as well as up to 20,000 shillings to cover transportation costs.

As the majority of the participants did not read or write, an approved process was engaged that allowed a participant to provide a thumbprint on the consent document with an impartial witness present to sign as the witness of the consent. When a participant was able to read and write, they would append a signature on their own. Participants were informed that an audio recording would be made of the interview, and they were additionally notified of their ability to withdraw from the study at any time, as well as their ability to pause the study process if they wanted a break. A trained local psychologist who spoke the local Rukiga language was made available on the research team in case support was needed to those participating given the nature of the topic that carried the chance of being distressing to some participants. One participant who initially agreed to participate in the study decided afterwards not to proceed with participation. The reason given was that they decided they did not want the relevant memories back because they knew, even if they shared their stories, that they were not going to have a chance to get those things they wanted back from the Forest. No other participant refused participation or withdrew from the study.

Interview data collection

Semi-structured interviews were carried out in-person by two local research assistants (SN, RM) from August to November 2022, and the interviews lasted between 45 and 90 minutes. The local research assistants both had a bachelor's level of education and between 3 and 5 years of experience working in qualitative research in Uganda. The research assistants had no prior relationships with the participants. Three days of supplementary training was provided to the research assistants prior to the onset of the participant interviews to ground them in the study procedures. Interview debriefs were also done periodically throughout the data-collection phase with the wider research team [22]. These debriefs were used to refine the interview process as needed, and to situate a reflexive process where assumptions and transferability were openly discussed along the way to ensure a grounding in the data itself. Each of the interviews was audio recorded with additional memos being taken to ensure appropriate reflection of the data being gathered. Interviews were carried out in the respective community gathering house that was available in each settlement; however, a few interviews were carried out in the homes of participants who were not very mobile and made the specific request to participate from home. An additional research lead who had an advanced knowledge or "Elder's level" of the local language remained on standby (SK) for the interviews in case some participants spoke words that were not understandable to the research assistants (SN, RM). If a word or concept was unclear, the research team would first seek further clarification of the word from the participant. If the translation still was not clear to the research team, then the advanced language speaker (SK) would be called for clarification before proceeding with the interview.

Interview guides were originally developed in English and then were translated to the local Rukiga language by a group of three study team members (SK, HN, JN). However, it took a number of translation iterations to ensure the translation to the local Rukiga language had the intended meaning [23]. Some English words do not translate into the Batwa language (e.g., mental health), so a free modulation translation approach was used to support the process [23]. In addition, at least one cyclical iteration of translation was engaged where the words were first translated from English to the local Rukiga language, and then again from the local Rukiga language to English to see if the same essential meanings were preserved. Interview questions were originally formulated with local community members using prior survey data that helped inform relevant areas of inquiry. Participants were asked open-ended questions

with the space to expand or delve into areas they felt were important to share. Participants were asked such questions about their relationship to the Forest, what they missed from the Forest, how being forcibly removed from the Forest affected them, and how often they thought about the loss of the Forest.

Data analysis

Interviews were transcribed verbatim into the local Rukiga language and then translated into English by a hired transcriber with verification done by additional research team leads (SK, HN). Transcripts were further anonymized to ensure that any local community attributes were removed (e.g., name of community buildings, organizations, etc) given the close-knit community and the need to ensure no identifying information was present. Interview transcripts were then uploaded into NVivo (Release 1.3) qualitative software for further analysis. Basic demographic data was additionally matched within the qualitative software to the interview transcript including the category of participant: 1) those who lived in the Forest before the forced land eviction (e.g., current Elders); 2) those who were born after the forced land eviction (i.e., lived during the eviction as a young person); and 3) those born after the forced land eviction). Thematic analysis was carried out on the interview transcripts according to Braun and Clarke (2006, 2019) [24, 25]. The progression of coding was tracked through analytic folders associated with the initial systematic coding of the data, the generation of initial themes from the initial codes, and then the refining, defining, and naming of themes to keep an audit trail [24, 25]. One author was involved in the preliminary coding (NR) with a second author brought in for discussion and refining of the codes and themes (SK). Data saturation was reached when no new themes were identified from the data [26].

Results

> "*We miss some things in the forest. . .our lives have never been the same ever since we left. . .*" *(ID 460)*

Nineteen semi-structured interviews were completed with nine self-identifying adult males and ten females represented in the sample. Participants ranged in age from 21 to 107 years of age with the majority having little to no 'Euro-western'-based education ($n = 17$). Eleven participants had previously lived in the Forest as adults, five as young people, and three were born after the forced eviction process. Participants were represented from all the main Batwa settlement areas in southwestern Uganda. The majority of the participants stated that they think about the loss of their land and the loss of their traditional foods several times a day, which was cross-cutting across age groups, gender, and geographic location.

There were four overarching themes identified from the data including: 1) Our love and connection with the Forest; 2) What was left in the Forest when we were evicted; 3) What eviction from the Forest did to us as Batwa Peoples; and 4) Batwa Peoples Landback and returning to the Forest. Relevant themes and sub-themes are listed in *Table 1*.

Our love and connection with the Forest ('Ihamba)

> "*We loved the forest so much.*" *(ID 453)*

Most of the participants reflected on the aspects they loved about the Forest, and what their connections to the Forest were ($n = 17$): "I have a connection with the forest because all my life

Table 1. Main themes and categories identified from the data.

Themes	Sub-themes
Our love and connection with the Forest	• n/a
What was left in the Forest when we were evicted	• Our foods and medicines from the Forest<br>• Our Ancestors<br>• Our animal friends<br>• Our Batwa spirituality
What eviction from the Forest did to us as Batwa Peoples	• Forced removal and disconnection from the Forest<br>• Suffering and many hardships<br>• Sadness and a lack of happiness<br>• The loss of our traditional foods and medicines<br>• Changes to and loss of our Batwa culture and ways of life<br>• Disrupted family ties and assimilation with non-Batwa Peoples and cultures<br>• Decreased health and increased death in our communities
Batwa Peoples Landback and returning to the Forest	• n/a

https://doi.org/10.1371/journal.pgph.0002129.t001

I want to be in the forest, because what I used to get from the forest is not what I get in the village . . .in the forest I would have my peace" (ID 469). Many saw the Forest as their home: "It is our old home, we love it. We loved the forest so much and we are sad that we left" (ID 453). Some participants reminisced about how ". . .the forest took care of us, we wish we would go back and stay" (ID 459). "It used to be our home and we ate without much struggle" (ID 460), and "I would be very happy, I would be even jumping if they told me that they are taking me back to my forest of birth" (ID 462). "The forest is more important than anything else because whatever I want and whatever sustained us as Batwa is in that forest" (ID 464). Several participants reflected on the foods they loved from the Forest, and that ". . .we had peace when we used to eat" (ID 467) the foods from the Forest.

What was left in the Forest when we were evicted

> "*We stayed in the forest most of the time and entirely survived on the foods and herbs from the forest.*" *(ID 457)*

All of the participants described things that were left in the Forest when they were evicted. The most common thing mentioned was the *foods and medicines* that were left in the Forest (n = 19). Of all words stated across the interview transcripts, four of the top five-word frequencies mentioned were related to food, including: 1. eat (172 mentions), 2. food (149 mentions), 3. meat (145 mentions), 4. honey (109 mentions). Various kinds of meat, honey, and yams as well as medicines to treat many kinds of ailments were left behind and are now inaccessible to the Batwa community for use. Some participants stated that ". . .we cannot be allowed to go back to look for these things. . ." (ID 464) due to the risk to self and safety; "I was recently imprisoned because I went to pick herbs from the park" (ID 454).

Several participants spoke about the *Ancestors* who were left behind in the Forest (n = 7): "In the forest we left there our great-grandparents and grand-grandparents because when they died that's where they were buried" (ID 466), ". . .we only left there the dead, so many of them" (ID 456). The *animal friends* were also left behind (n = 10), including both those that were received as food, and those that Batwa co-existed with in peace. Many participants referred specifically to leaving the "Gorillas. . .our friends. . ." (ID 453).

Part of the *Batwa spirituality* was also noted to be left in the Forest (n = 14): ". . .it's only Nyabingi that remained in the forest" (ID 467). "Yes, we did leave there Nyabingi, our God"

(ID 453), ". . .that kind of worship was left in the forest when we came here" (ID 464), and ". . .our prayers used to work a lot. . ." (ID 470). Participants talked about traditional Batwa spiritual practices and beliefs that were followed when living within the Forest. Collective spirituality was referenced where community members ". . .prayed as a group" (ID 454), however, ". . .we never had churches in the forest" (ID 460), instead prayers and offering were carried out in special caves, in small huts, or in trees within the Forest.

What eviction from the Forest did to us as Batwa peoples

All participants described various effects on their lives and the lives of their community from being forcibly evicted from the Forest (n = 19) as "we became squatters in people's land" (ID 466). Seven sub-themes were identified in this section including the *forced removal and disconnection from the Fores*t experienced by participants (n = 18). Participants shared memories or stories about the process of eviction and the disconnection from the Forest that is now a reality. "We were forcibly removed, beaten, and forced out" (ID 460); "they evicted us with guns" (ID 469), and ". . .we are no longer allowed to enter the forests" (ID 453)–"this was our home and we were chased out of it (ID 459). "Since I don't have any other place, I have to live where I am" (ID 463), however, the "taking of our forest makes us miserable" (ID 458).

As a consequence of the forced removal, one of the most common sub-themes was the *suffering and many hardships* that have occurred since the Land eviction (n = 19). "You are never at peace, always suffering with this and that (ID 458), and ". . .the Batwa suffered so much. . .they don't have much in the way of money and material possessions. . ." (ID 459)–"our lives have never been the same ever since we left" (ID 460). The abrupt change from having access to traditional foods in the forest to being stripped from these same food sources was noted to be taking a toll. In some cases, ". . .in seven days, I can fail to get food 4–5 days and spend the whole day without eating anything" (ID 462) ". . .because there is no food" (ID 467). There was also evidence of hopelessness with the situation amongst some participants, with one noting that "I can't do anything to fight for myself. . . the situation we are in is for surviving." (ID 463).

The suffering and hardships reported by participants clearly resulted in the sub-theme of *sadness and a lack of happiness* (n = 16). Participants shared the effects of the eviction on their overall mental wellbeing. "I don't get happy but rather sad because I think about what they took from us" (ID 454). . ."we loved the forest so much and we are sad that we left" (ID 453). ". . .It is sad being chased from your home" (ID 458), and "I feel very bad about it" (ID 455).

Another sub-theme was the effects of *the loss of traditional foods and medicines* on individuals and the community in general. Participants shared about the food and medicines they miss and that they can longer access because of the Land eviction. "The bush meat is like omugaju [rare/special scent], it is very sweet. I think about it a lot because there is no one who can never think about what they used to eat" (ID 467). "I have not eaten anything since morning, but would I be this hungry if I was in the forest? Everything is negative (ID 471), and "I miss the foods we used to eat and our way of life" (ID 453). One participant shared about their grandfather's response to the loss of the foods from the Forest: ". . .his heart remains in the forest thinking about the yams because he can't eat other foods and get satisfied. Sometimes when he thinks about it, he can spend a week without eating" (ID 468). The use of traditional medicines was also commonly mentioned as ". . .we miss our strong medicines that we used to take while in the forest, because every time we would take the medicines we would be strong and happy" (ID 470).

An additional sub-theme that was cross-cutting across all the interviews was the *changes and loss of our Batwa culture and ways of life* (n = 19). Participants described their cultural

practices before the eviction and how drastically things have changed in their way of life since the eviction. "The culture is not there. So basically, there may not be Batwa in the future because even our children don't have peace" (ID 469). . ."I cannot forget how I lived" (ID 454). Several participants mentioned missing materials to make traditional crafts from the Forest: "I miss those things we would get from there [the Forest]. . .such as baskets, those things to use for crafting (ID 466). There was a clear sense of loss in the younger generation who never had the opportunity to live in the Forest themselves. "I kept thinking about my parents and how we lived" (IDI 459). . ."We the young people have missed the [Batwa] culture" (ID 461).

Amplifying the cultural loss was the sub-theme of *disrupted family ties and assimilation with non-Batwa Peoples and cultures* (n = 12). Participants talked about the forced assimilation into non-Batwa communities and the disruption to Batwa family and cultural ties that occurred as a result of the eviction. "Ever since we were evicted from the forest, many Batwa died, and we were disintegrated. Some went to Congo, others to Rwanda, many died and perished" (ID 454). "There is an old man. . .he says that assimilation was hard. . .especially mixing with people whose culture they hardly knew" (ID 458). There were many participants who noted concerns from losing not only the culture and language due to assimilation but also themselves as a people. "Intermarriages have happened and in the near future, if the trend continues, I think we may not be there anymore" (ID 461), ". . .so, in the future, the Batwa will be few or will not exist at all" (ID 463).

A last notable sub-theme was *decreased health and increased death in our communities* (n = 15). Participants talked about how there was very little illness and death before the forced Land eviction and about how their peoples have had higher levels of sickness and death since then. "We used to not die very much, but when we left, we started to die. . .I think it is because of the food changes and environment" (ID 453). Upon having to leave the Forest, many people "got sick, especially our Elders" (ID 454), and ". . .they never used to die a lot like today" (ID 460). "When we left the forest, we started suffering and struggling with diseases because we could not get the local medicines we used to get from our forest of birth" (ID 462). "There was nothing like someone is sick and they have died. . .We had our local medicine that used to fight for us (ID 467). There was even mention of one's child dying during childbirth, ". . .though in the forest, women would give birth normally without complications" (ID 466). "We never used to die while in the forest because we had our local medicine there and we used to live longer" (ID 470), in fact, ". . .someone would grow very old to the extent of the body starting to peel and that's when they would die. Young people would never die" (ID 467).

Batwa peoples Landback and returning to the Forest ('Ihamba)

". . .every time I think about the forest, my heart feels like going back." (ID 471)

Participants described the desire for Landback, a term used to place "Indigenous lands back into Indigenous hands" [27], as well as reflections on returning to the forest (n = 18): "I would be very happy, I would be even jumping if they told me that they are taking me back to my forest of birth" (ID 462). Many participants understood clearly that the Forest is theirs and is their home. "Me I know the forest is mine. . .Our grandparents told us that the forest is ours" (ID 458). "They talk about how the forest belonged to us, how they lived in it, and that it should be given to us again" (ID 459). Several participants wanted to have a piece of the Forest back so they could teach their children their traditional ways. "I propose that they give me a small part of the forest and I teach my children things of the past (ID 454); with the younger generation also stating the desire ". . .to learn about our culture, who we are, and where we

came from" (ID 461). "If I had a voice that could be heard, I would suggest they give us part of the forest" (ID 469). Although there were many participants who thought it would be difficult, there was some stated hope that the Forest would one day be returned to the Batwa despite the obstacles. "I think the government will return our property" (ID 460). Many participants stated that if they had the chance to go back to the Forest they would.

> "*The responsible people should save us from this suffering and fight for us so that we can go back to the forest.*" *(ID 462)*

Discussion

> *"I tell them that when you grow up. . .make sure you fight to get back the forest." (ID 462)*

From the nineteen semi-structured interviews carried out for this study, four overarching themes were identified from the data gathered including: 1) Our love and connection with the Forest; 2) What was left in the Forest when we were evicted (including four sub-themes); 3) What eviction from the Forest did to us as Batwa Peoples (including seven sub-themes); and 4) Batwa Peoples Landback and returning to the Forest ("Indigenous lands back into Indigenous hands" [27]). The individual and community consequences of Land loss caused by forced eviction of the Batwa Indigenous Peoples in Uganda runs deep and across generations in the region.

Solastalgia itself can be "both natural and artificial" [12]; however, forced land eviction (i.e., artificial solastalgia) from conservation activities has received little to no coverage in the research literature on the topic until this current study. Further to this, out of twenty-nine papers identified in a recent scoping review on solastalgia, nearly all examined solastalgia "through a western worldview or through multiple worldviews, rather than using an Indigenous worldview specifically" [28]. Despite this lack of Indigenous presence in the solastalgia literature, some of the themes brought out in our current study were identified in previous studies in other populations. For example, feelings of powerlessness, disruptions to feeling a sense of place and identity, and challenges to mental and general wellbeing have been articulated [29, 30]. In one additional study carried out with Indigenous Torres Strait Islanders on solastalgia from climate change, impacts identified included "feelings of sadness, worry, fear and distress, along with a declining sense of self, belonging and familiarity" [31]. Unique to our study was the profound sense of loss felt from losing access to the Batwa traditional foods and medicines with four of the top five-word frequencies mentioned being related to food, as previously noted.

In addition, solastalgia may be examined through the lens of previously identified long-term emotional consequences of colonial policies on Indigenous Peoples in other regions including: "historical loss", "intergenerational posttraumatic stress disorder", "historical trauma", and "historical grief" [32–36]. In North America, for example, Indigenous Peoples were forcibly removed from Lands, starved, and forbidden to practice their traditional cultural practices for generations, which has received increasing research and policy support, including Landback agendas [27, 37–40]. Despite the still very present funding and support deficits for Indigenous initiatives in North America, Indigenous Peoples in Africa have been particularly marginalized within research and policy spaces, with a complete lack of policy and legislative safeguards, including the non-existence of formalized Land treaties [1, 4, 5, 7, 8].

Forced Land evictions for conservation have not been exclusive to the Batwa Peoples, and they have not just been an instrument of past conservation practice. The Maasai Peoples of

northern Tanzania are currently again being evicted from their ancestral land "in the name of conservation" [41], with untold community impacts. With renewed international movements and calls for the "30 by 30" to be realized [42], which situates the desire to have 30% of Earth's land and sea areas conserved; Indigenous communities and rights advocates have stated clearly that this plan will lead to more human rights abuses around the globe [43]. The very real risk of continued and amplified mass evictions of Indigenous Peoples from their homelands has been largely ignored in policy debates surrounding the "30 by 30" agenda [44].

As an example, Batwa Peoples have even been arrested for picking their own traditional medicines [45] due to the militarization of the conservation agenda [46], with governments and conversation groups failing to uphold basic Human Rights and the Rights of Indigenous Peoples [5, 8, 9]. Despite international calls for the uplifting of Indigenous traditional ecological knowledge (TEK) as part of the solution to the climate and biodiversity crisis, Batwa Peoples have not been given the opportunity to pass down their mass amounts of ecological stewardship knowledge that has kept both the Forest and themselves healthy and well for generations.

Indigenous Peoples make up approximately 5% of the world's population, occupy 22% of the world's surface, yet actively steward 80% of the remaining biodiversity on the planet [47]. In less than a generation more of forced Land displacement of Batwa Peoples, hundreds of years of important ecological and Forest stewardship knowledge may be lost for practical community use [48], the traditional Land-based language that is the blueprint of that ecological knowledge may be lost [49], and most importantly, Batwa Peoples will not be given the opportunity to thrive as a community [50–52]. A large proportion of the current Batwa community has never lived in the Forest, instead growing up on the fringes of the Forest with enforcement officers not allowing them in. All of this loss due to Westernized conservation agendas that have failed to acknowledge the importance of Indigenous Peoples and their knowledges for the survival of us all as a human species [1, 3, 4, 5].

The Fiscus tree (*Ficus carica*) is a tree in the Forest that has significant importance to Batwa Peoples. From being at the center of their spirituality, to the tree's uses as medicine, clothing, and shelter, the Fiscus tree is still waiting in the Forest to have its relatives the Batwa back to caretake it (see *Fig 1*). Indigenous Peoples have reciprocal relationships to the environment, with all knowledge coming from the Land around them [53], yet the sheer amount of sadness and loss that was represented in the research data from this study is hard to ignore. The Fiscus tree was one of the plant relatives left in the Forest in addition to all the other plant and animals relatives the Batwa have co-existed with and depended on for centuries—all left behind. Even the graves of the Batwa ancestors were left—relatives they can no longer visit. Despite these imposed hardships, Batwa Peoples are resilient, beautiful people who deserve to have their voices and the voice from their Forest be heard.

"*The forest took care of us, we wish we would go back and stay.*" *(ID 459)*

Limitations

The study made a strong effort to ensure broad representation from many Batwa communities, genders, and ages in the Kanungu district. Despite this, the data presented may not be fully representative of all members of all Batwa districts in the region. Due to data saturation occurring, and how cross-cutting the findings were, we are confident that the data gives good insights into the main important elements of this topic area in the district. Qualitative research allows Indigenous Peoples to share their own perspectives in a way they feel most comfortable; however, qualitative research results cannot be assumed to be generalizable to other

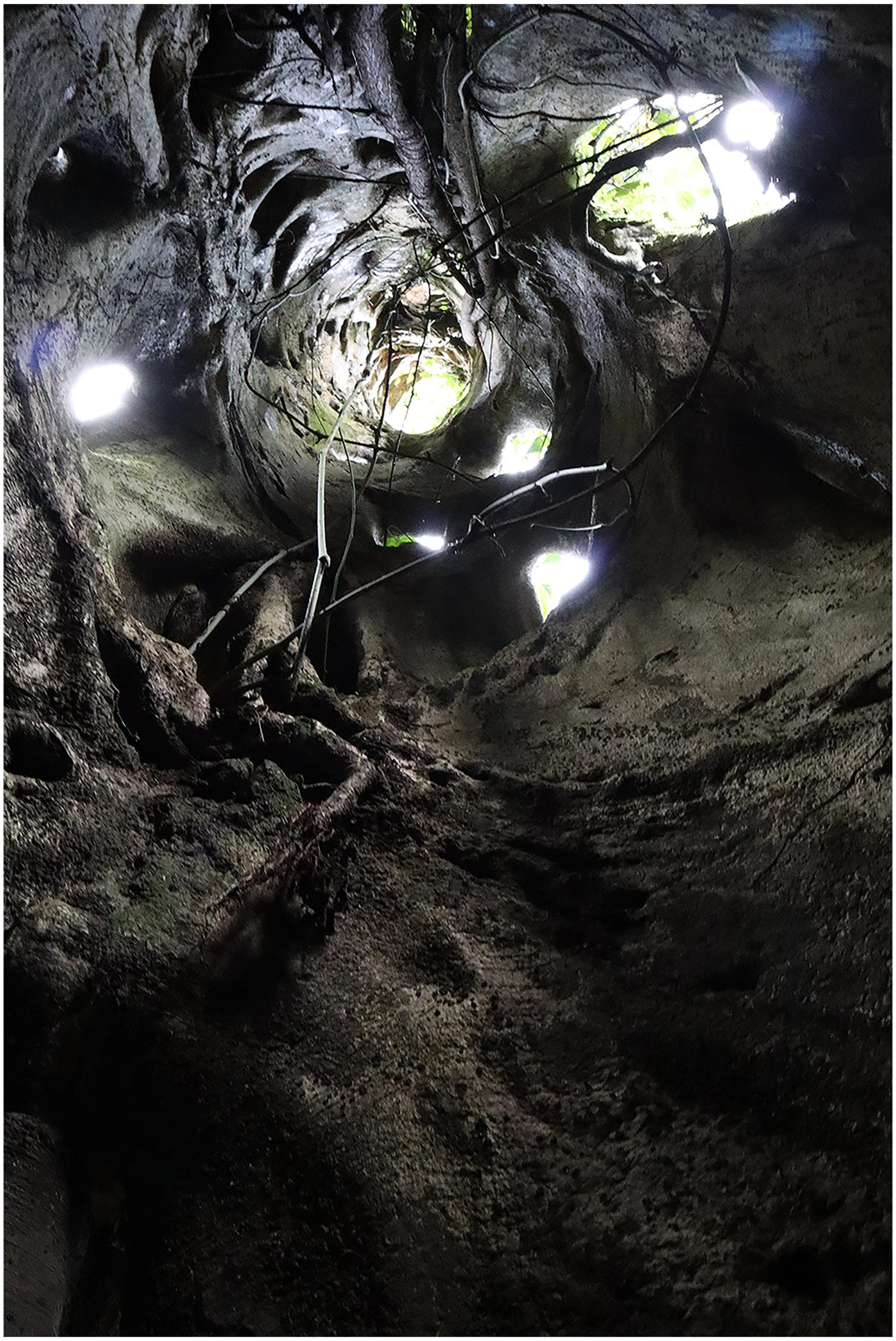

Fig 1. The sacred Fiscus tree (*Ficus carica)* of the Batwa peoples. *Republished from an unpublished collection under a CC BY license, with permission from Invisible Flock, original copyright 2021.

https://doi.org/10.1371/journal.pgph.0002129.g001

Indigenous communities that have faced forced Land evictions in Africa or in other locations. Due to the lack of published research examining solastalgia in the context of Indigenous Peoples and forced Land evictions in Africa, it is difficult to assume any potential transferability to other contexts. Due to some of the similarities identified in research with Indigenous Peoples on other continents in the context of "historical loss" [34, 54] and "historical trauma" [55–57], however, we believe that there may be some relevance of the findings in this current study to

other Indigenous communities in Africa that is worthy of further research and exploration. We advocate for more Indigenous-led research and policy agendas in Africa around Land evictions and its related impacts on communities with a focus on re-establishing communities to their traditional ancestral homelands.

Conclusion

> *"The forest is so important to me." (ID 471)*

Despite several conservation groups, non-governmental organizations, and local governments citing impressive collaborative conservation work with local communities in Africa, the realities on the ground for Batwa communities in Uganda were found in this study to be a far cry from outwardly portrayed narratives. The experience and state of solastalgia, artificially induced from forced Land eviction for conservation, have been an underappreciated and unacknowledged element of Indigenous Peoples wellbeing and ability to thrive in Africa and around the globe. There is clearly a lack of conservation voices standing up for Batwa and other communities to ensure that colonial and paternalistic systems that have continued to marginalize and exclude Indigenous Peoples from decision making and funding availability is rectified. Batwa Peoples are forest ecology and stewardship experts, knowledge holders in their own right, with keen political agendas for their community [58]. In addition, the Batwa have their own decolonial research frameworks that need to be honoured in any future research work with and by the community. As movement towards the global "30 by 30" conservation agenda occurs, we urge researchers, policy makers, and leaders to listen to the voices of Indigenous Peoples like the Batwa with a key focus on Landback, and movement towards a clearer understanding and appreciation of the impacts of Western conservation agendas on Indigenous Peoples globally.

Supporting information

S1 File. Inclusivity in global research.
(PDF)

Acknowledgments

We would like to formally acknowledge *Ihamba* (the Forest) whose own voice and presence has been consistently ignored by those making decisions *for* Batwa Peoples and not *with* Batwa Peoples. The Forest is the Batwa Peoples and the Batwa Peoples are the Forest. We would therefore like to formally acknowledgment the Batwa Peoples and community that supported this work from start to finish, with additional special thanks to Tumuhimbise Ishamael. We would also like to thank Invisible Flock and Land Body Ecologies for their support of this project.

Author Contributions

Conceptualization: Sylvia Kokunda, Haven Nahabwe, Jeremiah Nahamya, Samrawit Gougsa.

Data curation: Sylvia Kokunda, Haven Nahabwe, Nicole Redvers.

Formal analysis: Nicole Redvers.

Funding acquisition: Sylvia Kokunda, Samrawit Gougsa.

Investigation: Sylvia Kokunda, Haven Nahabwe, Jeremiah Nahamya, Samari Niwamanya, Ronald Mazirwe.

Methodology: Sylvia Kokunda, Haven Nahabwe, Elizabeth Kemigisha, Nicole Redvers.

Project administration: Sylvia Kokunda, Haven Nahabwe, Samrawit Gougsa, Nicole Redvers.

Resources: Samrawit Gougsa.

Software: Nicole Redvers.

Supervision: Sylvia Kokunda, Haven Nahabwe, Nicole Redvers.

Writing – original draft: Nicole Redvers.

Writing – review & editing: Sylvia Kokunda, Haven Nahabwe, Jeremiah Nahamya, Samari Niwamanya, Ronald Mazirwe, Samrawit Gougsa, Elizabeth Kemigisha, Nicole Redvers.

References

1. Dowie M. Conservation Refugees. 2010 [cited 2023 March 18]. In: Cultural Survival [Internet] Available from: https://www.culturalsurvival.org/publications/cultural-survival-quarterly/conservation-refugees
2. Ahebwa WM, van der Duim R, Sandbrook C. Tourism revenue sharing policy at Bwindi Impenetrable National Park, Uganda: a policy arrangements approach. J. Sustain. Tour. 2012; 20(3): 377–394.
3. Zaninka P. The impact of (forest) nature conservation on Indigenous peoples: the Batwa of south-western Uganda. A case study of the Mgahinga and Bwindi Impenetrable Forest Conservation Trust. In: Forest Peoples Programme, Nelson J, Hossack L. From principles to practice: Indigenous peoples and protected areas in Africa. Utrecht, Netherlands: Drukkerij Zuidam & Uithof; 2001. pp. 165–194.
4. Rodriguez ND. Consequences of conservation-induced displacement: A case study with Batwa participants in Southwest Uganda. Master of Arts, University of Colorado Colorado Springs. 2021. Available from: https://www.proquest.com/docview/2562283851?pq-origsite=gscholar&fromopenview=true
5. Mukasa N. The Batwa Indigenous People of Uganda and Their Traditional Forest Land: Eviction, Non-Collaboration and Unfulfilled Needs. Int. Indig. Policy J. 2014; 24: 1–8.
6. Mbarara University of Science and Technology, Gulu University Constituent College, Makerere University. A desk review report of the voices and rights of the Batwa, the IK, and the Tepeth Indigenous Peoples and the trafficking in persons in Karamoja, Uganda. 2020 [cited 2023 March 18]. Available from: https://pdf.usaid.gov/pdf_docs/PA00X3W5.pdf (accessed 18 March 2023).
7. Roberts K. Understanding the Marginalized Indigenous Batwa People of Southwestern Uganda: Co-creation Event Report. 2022 [cited 2023 March 18]. Available from: https://laserpulse.org/publication/understanding-the-marginalized-indigenous-batwa-people-of-southwestern-uganda-co-creation-event-report/
8. Domínguez L, Luoma C. Decolonising Conservation Policy: How Colonial Land and Conservation Ideologies Persist and Perpetuate Indigenous Injustices at the Expense of the Environment. Land 2020; 9 (3): 65.
9. United Nations. United Nations Declaration on the Rights of Indigenous Peoples. 2007 [cited 2023 March 20]. Available online: https://social.desa.un.org/issues/indigenous-peoples/united-nations-declaration-on-the-rights-of-indigenous-peoples
10. Survival. Uganda: Batwa "Pygmy" faces prison in the name of conservation'. 2017 [cited 2023 March 20]. Availabe from: https://www.survivalinternational.org/news/11567
11. Bako JP. Walking the talk: are land evictions in Uganda in like with human rights standards? LLM (Multidisciplinary Human Rights), University of Pretoria. 2009. Available from: https://repository.up.ac.za/handle/2263/26966
12. Albrecht G. 'Solastalgia': a new concept in health and identity. PAN: philosophy activism nature. 2005; 3:44–59. https://doi.org/10.4225/03/584f410704696
13. Roach P, McMillan F. Reconciliation and Indigenous self-determination in health research: A call to action. PLOS glob. public health. 2022; 2(9): e0000999. https://doi.org/10.1371/journal.pgph.0000999 PMID: 36962858
14. Lock M, McMillan F, Bennett B, Martire JL, Warne D, Kidd J, et al. Position statement: Research and reconciliation with Indigenous peoples in rural health journals. Australian Journal of Rural Health. 2022; 30(1): 6–7. https://doi.org/10.1111/ajr.12834 PMID: 35043514

15. Marsden N, Star L, Smylie J. Nothing about us without us in writing: aligning the editorial policies of the Canadian Journal of Public Health with the inherent rights of Indigenous Peoples. Can. J. Public Health. 2020; 111(6): 822–825.
16. Ruckstuhl K. Trust in Scholarly Communications and Infrastructure: Indigenous Data Sovereignty. Front Res Metr Anal. 2021; 6: 752336. https://doi.org/10.3389/frma.2021.752336 PMID: 35098013
17. Tsosie KS, Yracheta JM, Kolopenuk JA, Geary J. We Have "Gifted" Enough: Indigenous Genomic Data Sovereignty in Precision Medicine. Am J Bioeth. 2021; 21: 72–75.
18. Dalton R. Tribe blasts 'exploitation' of blood samples. Nature. 2002; 420(6912): 111. https://doi.org/10.1038/420111a PMID: 12432350
19. Cull I, Hancock RLA, McKeown S, Pidgeon M, Vedan A. Pulling Together: A Guide for Front-Line Staff, Student Services, and Advisors. In: Decolonization and Indigenization. Victoria, BC, Canada: Pressbooks; 2019. pp. 1–99.
20. Brennan F, Ungunmerr-Baumann, MR. Reverencing the Earth in the Australia Dreaming. The Way. 1989; 29: 38–45.
21. O'Brien BC, Harris IB, Beckman TJ, Reed DA, Cook DA. Standards for Reporting Qualitative Research: A Synthesis of Recommendations. Acad Med. 2014; 89(9): 1245–1251. https://doi.org/10.1097/ACM.0000000000000388 PMID: 24979285
22. McMahon SA, Winch PJ. Systematic debriefing after qualitative encounters: an essential analysis step in applied qualitative research. BMJ Glob Health. 2018; 3(5): e000837. https://doi.org/10.1136/bmjgh-2018-000837 PMID: 30233833
23. Putranti A. Modulation: A Translation Method to Obtain Naturalness in Target Language Texts. J. Lang. Lit. 2018; 18: 98–101.
24. Braun V, Clarke V. Using thematic analysis in psychology. Qual. Res. Psychol. 2006; 3(2): 77–101.
25. Braun V, Clarke V. Reflecting on reflexive thematic analysis. Qual. Res. Sport Exerc. Health. 2019; 11 (4): 589–597.
26. Guest G, Bunce A, Johnson L. How Many Interviews Are Enough?:An Experiment with Data Saturation and Variability. Field Methods. 2006; 18(1): 59–82.
27. NDN Collective. LandBack. Availabe online: https://landback.org/ (accessed on 21 March 2023).
28. Galway LP, Beery T, Jones-Casey K, Tasala K. Mapping the Solastalgia Literature: A Scoping Review Study. International Journal of Environmental Research and Public Health. 2019; 16(15): 2662. https://doi.org/10.3390/ijerph16152662 PMID: 31349659
29. Albrecht G, Sartore GM, Connor L, Higginbotham N, Freeman S, Kelly B, et al. Solastalgia: The Distress Caused by Environmental Change. Australasian Psychiatry. 2007; 15(1_suppl): S95–S98. https://doi.org/10.1080/10398560701701288 PMID: 18027145
30. Ellis NR, Albrecht GA. Climate change threats to family farmers' sense of place and mental wellbeing: A case study from the Western Australian Wheatbelt. Soc Sci Med. 2017; 175: 161–168. https://doi.org/10.1016/j.socscimed.2017.01.009 PMID: 28092757
31. McNamara KE, Westoby R. Solastalgia and the Gendered Nature of Climate Change: An Example from Erub Island, Torres Strait. EcoHealth. 2011; 8: 233–236. https://doi.org/10.1007/s10393-011-0698-6 PMID: 21882055
32. Duran E, Duran B. Native American postcolonial psychology. Albany, New York, United States: Suny Press; 1995. pp. 1–246.
33. Brave Heart MYH, DeBruyn LM. The American Indian holocaust: Healing historical unresolved grief. Am. Indian Alsk. Native Ment. Health Res. 1998; 8(2): 60–82. PMID: 9842066
34. Whitbeck LB, Adams GW, Hoyt DR, Chen X. Conceptualizing and measuring historical trauma among American Indian people. Am. J. Community Psychol. 2004; 33(3–4): 119–130. https://doi.org/10.1023/b:ajcp.0000027000.77357.31 PMID: 15212173
35. Brave Heart MYH. Gender differences in the historical trauma response among the Lakota. J Soc Policy. 1999; 10(4): 1–21. https://doi.org/10.1300/J045v10n04_01 PMID: 10538183
36. Brave Heart MYH. The return to the sacred path: Healing the historical trauma and historical unresolved grief response among the Lakota through a psychoeducational group intervention. Smith Coll. Stud. Soc. Work. 1998; 68(3): 287–305.
37. Pieratos NA, Manning SS, Tilsen N. Land back: A meta narrative to help Indigenous people show up as movement leaders. Leadership. 2021; 17(1): 47–61.
38. Brown NA. Continental Land Back: Managing Mobilities and Enacting Relationalities in Indigenous Landscapes. Mobilities. 2022; 17(2): 252–268.

39. Fisk JJ, Jacobs LA, Russo BUK, Meier E, Nakachi A, Spencer KKP, et al. Cultivating sovereignty in parks and protected areas: Sowing the seeds of restorative and transformative justice through the #LANDBACK movement. Parks Stewardship Forum. 2021; 37(3): 517–527.

40. Spang KC. #Landback: Northern Cheyenne to Reclaim Sovereignty. Tapestries: Interwoven voices of local and global identities. 2023; 12(1): 1–14.

41. Nyeko O, Nnoko-Mewanu J. Tanzania's Eviction of Maasai Pastoralists Continues. 2023 [cited 2023 March 23]. Available from: https://www.hrw.org/news/2023/02/02/tanzanias-eviction-maasai-pastoralists-continues#:~:text=In%20June%2C%20residents%20of%20Loliondo,in%20the%20name%20of%20conservation

42. Rowling M. Drive for goal to protect 30% of planet by 2030 grows to 50 nations. 2021 [cited 2023 March 23]. In: Human Rights Watch [Internet]. Available online: https://www.reuters.com/article/global-environment-nature-climate-change-idAFL8N2JM2YM

43. Mukpo A. As COP15 approaches, '30 by 30' becomes a conservation battleground. 2021 [cited 2023 March 23]. In: Mongabay [Internet]. Available from: https://news.mongabay.com/2021/08/as-cop15-approaches-30-by-30-becomes-a-conservation-battleground/

44. Geisler C, De Sousa R. From refuge to refugee: the African case. Public Adm Dev. 2001; 21: 159–170.

45. Kokunda S. "Born from the Forest". 2021 [cited 2023 March 25]. In: Medium [Internent]. Available online: https://medium.com/@landbodyecologies/born-from-the-forest-de3debc0a318

46. Ramutsindela M, Matose F, Mushonga T. The violence of conservation in Africa: state, militarization and alternatives. Cheltenham, United Kingdom: Edward Elgar Publishing; 2022. pp. 1–272.

47. Trewin B, Damian Morgan-Bulled D, Cooper S. Climate. 2021 [cited 2023 March 26]. In: Australia State of the Envirnment [Internet]: Available from: https://soe.dcceew.gov.au/climate/management/national-and-international-frameworks#-cli-21-figure-21-indigenous-peoples-and-the-environment

48. Tang R, Gavin MC. A Classification of Threats to Traditional Ecological Knowledge and Conservation Responses. Conserv. Soc. 2016; 14(1): 57–70.

49. Redvers N, Menzel K, Ricker A, Lopez-Carmen VA, Blondin B. Expanding the scope of planetary health education: The International Decade of Indigenous Languages. Lancet Planet. Health. 2023; 7(1): e4–e5. https://doi.org/10.1016/S2542-5196(22)00140-1 PMID: 36608947

50. Ahmed F, Zuk AM, Tsuji LJS. The Impact of Land-Based Physical Activity Interventions on Self-Reported Health and Well-Being of Indigenous Adults: A Systematic Review. Int. J. Environ. Res. Public Health. 2021; 18(13): 7099. https://doi.org/10.3390/ijerph18137099 PMID: 34281031

51. Biddle N, Swee H. The Relationship between Wellbeing and Indigenous Land, Language and Culture in Australia. Aust Geogr. 2012; 43(3): 215–232.

52. Ward LM, Hill MJ, Antane N, Chreim S, Olsen Harper A, Wells S. "The Land Nurtures Our Spirit": Understanding the Role of the Land in Labrador Innu Wellbeing. Int. J. Environ. Res. Public Health. 2021; 18 (10): 5102.

53. Redvers N, Faerron Guzmán CA, Parkes MW. Towards an educational praxis for planetary health: a call for transformative, inclusive, and integrative approaches for learning and relearning in the Anthropocene. Lancet Planet Health. 2023; 7(1): e77–e85. https://doi.org/10.1016/S2542-5196(22)00332-1 PMID: 36608953

54. Whitbeck LB, Walls ML, Johnson KD, Morrisseau AD, McDougall CM. Depressed affect and historical loss among North American Indigenous adolescents. Am. Indian Alsk. Native Ment. Health Res. 2009; 16(3): 16. https://doi.org/10.5820/aian.1603.2009.16 PMID: 20052631

55. Gone JP, Hartmann WE, Pomerville A, Wendt DC, Klem SH, Burrage RL. The impact of historical trauma on health outcomes for Indigenous populations in the USA and Canada: A systematic review. Am Psychol. 2019; 74(1): 20–35. https://doi.org/10.1037/amp0000338 PMID: 30652897

56. Smallwood R, Woods C, Power T, Usher K. Understanding the impact of historical trauma due to colonization on the health and well-being of Indigenous young peoples: a systematic scoping review. J. Transcult. Nurs. 2021; 32(1): 59–68. https://doi.org/10.1177/1043659620935955 PMID: 32567510

57. Pihama L, Reynolds P, Smith C, Reid J, Smith LT, Nana RT. Positioning historical trauma theory within Aotearoa New Zealand. AlterNative: An International Journal of Indigenous Peoples. 2014; 10(3): 248–262.

58. Ampumuza C, Duineveld M, van der Duim R. The most marginalized people in Uganda? Alternative realities of Batwa at Bwindi Impenetrable National Park. World Dev. Perspect. 2020; 20: 100267.
